# Supplementary material for: Atypical Hepatitis B Virus Serology Profile—Hepatitis B Surface Antigen-Positive/Hepatitis B Core Antibody-Negative—In Hepatitis B Virus/HIV Coinfected Individuals in Botswana
Source: Viruses. 2023 Jul 13;15(7):1544. doi: 10.3390/v15071544 (PMC10383918; doi:10.3390/v15071544)
Supplement: Supplementary file 1 [file viruses-15-01544-s001.zip › viruses-2486721-supplementary.pdf]

Supplemental Table S1: HBV viral load and ART regimen

		HBsAg+/Anti-HBc-		
	**TDF-containing**	**3TC-containing**	**Non-TFV, non-3TC containing**	**Total**
TND	3 (60)	1 (33.3)	1 (100)	**5 (55.6)**
<2000	1 (20)	0 (66.7)	0 (0)	**1 (11.1)**
≥2000	1 (20)	2 (50)	0 (0)	**3 (33.3)**
**Total**	**5**	**3**	**1**	**9**
		HBsAg+/Anti-HBc+		
TND	13 (27.1)	11 (34.4)	1 (33.3)	**25 (30.1)**
<2000	28 (58.3)	15 (46.9)	2 (66.7)	**45 (54.2)**
≥2000	7 (14.6)	6 (18.8)	0 (0)	**13 (15.7)**
**Total**	**48**	**32**	**3**	**83**

TDF; Tenofovir disoproxil fumarate, 3TC; lamivudine, HBsAg; hepatitis B surface antigen, Anti-HBc; hepatitis B core antibodies, TND; target not detectable
